# Synergetic Usage of Indigenous Communication Channels and ICTs for Sharing Vegetable Marketing Information: A Bivariate Probit Analysis From East Hararghe Zone, Ethiopia

**DOI:** 10.1155/tswj/4844995

**Published:** 2026-10-12

**Authors:** Getahun Million Agago, Getachew Shambel Endris, Chanyalew Seyoum Aweke

**Affiliations:** ^1^ School of Rural Development and Agricultural Innovations College of Agriculture and Environmental Sciences Haramaya University Maya Ethiopia haramaya.edu.et

**Keywords:** bivariate probit, Ethiopia, ICT, indigenous communication channels, information synergy, smallholder farmers, vegetable marketing

## Abstract

This study investigated the determinants of the synergistic use of indigenous communication channels (ICCs) and information and communication technologies (ICTs) for vegetable marketing information among smallholder farmers in East Hararghe Zone, Ethiopia. Using a mixed‐methods design and a multistage sampling approach, data were gathered from 385 farmers through structured interviews, focus group discussions, and key informant interviews. Data analysis applied descriptive statistics, a bivariate probit model, and thematic analysis. The bivariate probit results confirmed a statistically significant positive correlation between indigenous and modern channels (rho = 0.498, *p* < 0.05), demonstrating that farmers utilize these communication streams complementarily rather than as substitutes. This synergy enables smallholders to crossvalidate rapid ICT market updates with trusted local networks, mitigating transaction costs and information asymmetry to secure improved prices and income stability. Institutional factors emerged as primary drivers of joint usage: access to credit, mobile connectivity, and extension contact increased the probability of synergistic channel adoption by 30.4%, 26.2%, and 5.0%, respectively. Conversely, farm size, electricity, and formal education functioned primarily as prerequisites for standalone ICT adoption. Notably, older age favored traditional channels while constraining digital engagement because of lower digital self‐efficacy. Policy interventions should prioritize rural electrification, ICT‐integrated credit facilities, and age‐sensitive extension programs that strategically blend indigenous knowledge networks with digital tools to broaden market reach.

## 1. Introduction

Agriculture remains the fundamental cornerstone of economic development in sub‐Saharan Africa, serving as a primary pathway toward poverty alleviation and inclusive growth [1, 2]. In Ethiopia, the sector underpins the national macro economy, contributing approximately 32.4% to the gross domestic product (GDP), employing over 72% of the total labor force, and generating more than 75% of total foreign exchange earnings [3]. Because smallholder agricultural households drive over 90% of total crop output, providing them with timely, accurate, and relevant agricultural information is indispensable for increasing land productivity, strengthening rural food security, and stimulating broader rural‐urban economic linkages [4, 5].

Vegetable production, in particular, plays a vital role in creating employment and enhancing food security across Ethiopia′s diverse agroecological zones [6, 7]. Although vegetable marketing is highly profitable, average smallholder yields remain constrained by structural challenges, such as poor seed quality, severe pest outbreaks, and substantial postharvest losses estimated at 25%–40% of total production depending on commodity perishability [8–10]. These technical issues are severely compounded by inadequate market information systems, weak vertical marketing linkages, and the high biological perishability of fresh produce. In the East Hararghe Zone, characterized by limited extension contact and inadequate transport infrastructure, the lack of real‐time market data leaves smallholders particularly vulnerable to asymmetric pricing dynamics managed by commercial market intermediaries [11–13].

To alleviate these challenges, farmers require well‐organized access to both agricultural information sources and effective communication channels [14]. Although information and communication technologies (ICTs) such as mobile advisory services and internet platforms offer transformative potential to bridge real‐time information gaps, their adoption among smallholder farmers in rural Ethiopia remains constrained by structural digital barriers [15]. Empirical evidence indicates that although basic mobile phone ownership among rural households in eastern Ethiopia (specifically East Hararghe) has reached 65%–68%, actual utilization of ICT tools and internet‐enabled agricultural market services remains under 20%, with smartphone ownership staying below 15% in rural agrarian communities [15]. Recent field studies conducted across vegetable‐producing smallholders in Haramaya and Kombolcha Districts confirm that even though mobile phones and informal friends′ networks (friends, neighbors) are the top preferred channels across production seasons, mobile usage is overwhelmingly restricted to voice calls to query local traders and brokers [16]. Higher level ICT applications, such as SMS alerts, web‐based market applications, and smart digital platforms, suffer from negligible adoption because of erratic network infrastructure, high data costs, low digital literacy, and a lack of localized content [15]. Because these structural digital barriers prevent ICTs from acting as a standalone solution, rural communities in East Hararghe continue to rely heavily on indigenous communication channels (ICCs). These traditional, nonbureaucratic networks—including local social structures, informal market exchanges, village gatherings, and religious institutions—are culturally resonant, highly trusted, and virtually cost‐free [17]. However, although indigenous channels foster deep community trust, their slow dissemination speeds render them inadequate on their own for transmitting the fast‐changing price data required in highly perishable vegetable marketing [16, 18]. Consequently, there is a strong imperative to investigate the potential synergies between traditional social structures and mobile‐enabled information pathways.

The fundamental research problem is the absence of a deliberate and effective synergy between indigenous channels and ICTs, although existing literature widely acknowledges that no single channel can independently satisfy the complex information needs of smallholder farmers [19, 20]. Agricultural communication research in Ethiopia has largely treated these systems as independent or competing entities. Recent empirical studies continue to evaluate digital tools and indigenous networks in isolation rather than exploring their integrated potential [21, 22]. Consequently, there remains a critical empirical gap regarding how these distinct communication domains interact, and what specific demographic, socioeconomic, and institutional factors drive or hinder their integrated, concurrent usage.

This study addresses this gap by investigating the synergistic framework where ICT speed is validated by traditional indigenous trust. This dynamic is uniquely vital for vegetable marketing in the East Hararghe Zone (specifically Haramaya and Kombolcha Districts), where high crop perishability demands immediate marketing information, yet high market risk demands local verification.

By modeling this intersection through a bivariate probit framework, this study moves beyond traditional adoption literature to identify the precise determinants of joint‐channel synergy, thereby providing empirical insights to align regional extension policies with actual smallholder communication behaviors. To achieve this, the study fulfills the following specific objectives:

1. To characterize the demographic, socioeconomic, and institutional profiles of smallholder vegetable farmers in the Haramaya and Kombolcha Districts.
2. To identify and quantify the specific factors that determine the synergistic use of ICCs and ICTs for sharing vital vegetable marketing information.

## 2. Literature Review

### 2.1. Synergy

Synergy is defined as the functional integration of two or more systems to produce a collective effect greater than the sum of their individual contributions [23]. The concept of synergy is supported by the theory of system thinking [24, 25]. System thinking is a framework for understanding complex phenomena by examining the interconnections and interactions within a system. It has evolved significantly over time, influenced by diverse disciplines and thinkers, and today it plays a crucial role in addressing global challenges, such as climate change, health crises, and organizational complexity. In the context of this study, it refers to the joint utilization of ICCs and ICT‐based platforms for the exchange of agricultural marketing information among vegetable producers. The goal is not to replace ICCs with ICT channels or vice versa, but rather to create a synergistic usage that leverages the strengths of both channels [26].

### 2.2. ICCs

ICCs are enduring, socially regulated media forms that retain deep institutional legitimacy within rural communities despite the expansion of modern telecommunications [27]. Operating within community‐managed frameworks, ICCs utilize highly localized verbal and nonverbal structures, such as oral literature (*mammaaksa* and *hibboo*), informal socioinstitutions (*afoosha* and *ididir)*, religious institutions (churches and mosques), peer groups (guuzza), and farm visits (*waarii* or *dori*) [17, 18].

As emphasized by the Food and Agriculture Organization [28], these channels possess high systemic credibility because local communities retain complete ownership over the information architecture. However, from an information systems perspective, ICCs are structurally constrained by slow geographical diffusion speeds and localized boundaries, rendering them highly vulnerable to information asymmetry when confronted with dynamic, external macromarket fluctuations.

### 2.3. ICT

Conversely, ICTs encompass the hardware, software, and electronic infrastructure specifically mobile phones, radio, television, and SMS networks used to process, transmit, and retrieve agricultural data [28, 29]. In the context of sub‐Saharan digital advisory platforms (e‐extension), these tools alleviate market frictions by providing real‐time, site‐specific pricing data, localized weather forecasts, and direct buyer–producer linkages [30, 31]. Recent empirical meta‐analyses confirm that mobile phone–mediated networks significantly reduce agricultural market entry barriers and information search costs [32, 33]. Yet, in rural Ethiopia, the lone efficacy of these tools is strictly bottlenecked by high capital costs, language barriers, and a lack of contextualized, verified content, which frequently triggers user skepticism.

### 2.4. Marketing Information

Marketing information encompasses timely data on fluctuating farm gate and terminal prices, buyer quality standards, seasonal supply‐demand dynamics, and market destinations required by smallholder producers to navigate perishable product markets [34]. Studies in Ethiopia specifically examine how mobile phones, radio, and interpersonal networks are utilized by smallholder farmers for food crop and horticulture market information, highlighting their role in improving real‐time access to critical market data [35].

Beyond ICTs, ICCs remain vital for vegetable marketing among smallholders. Farmers often rely on word‐of‐mouth from neighbors, friends, local brokers (delalas), and community leaders for immediate price updates, market demand shifts, and trade negotiations. These traditional channels build deep trust and facilitate rapid, highly localized information exchange that is critical for managing perishable produce [16].

### 2.5. Operational Definition of Vegetable Marketing Information

In the context of this study, vegetable marketing information is operationally defined as the comprehensive, real‐time intelligence encompassing output prices, product quality standards, supply and demand volumes, and market locations exchanged and utilized by smallholder farmers across all stages from production to final sale, through the concurrent and synergistic use of both ICCs and ICTs.

Effective marketing is paramount for smallholder farmers in developing countries, especially for vegetable crops, which are highly perishable and demand immediate market intelligence. Real‐time access to market information is crucial for maximizing profitability and minimizing postharvest losses [36]. Traditionally, ICCs such as discussions with friends and neighbors, community meetings, local market agents, and word‐of‐mouth have facilitated market interactions, fostering trust and cultural relevance [37]. However, these channels often suffer from limited spatial reach, slow dissemination, and spatial information asymmetry, particularly across distant regional urban markets.

The rapid spread of ICTs, primarily basic mobile phones and radio, in rural Ethiopia has opened new avenues for farmers to bypass traditional information bottlenecks [38, 39]. Yet, mere ICT availability does not guarantee effective adoption or integrated use. The concept of communication synergy is therefore vital: a symbiotic relationship where ICCs and ICTs mutually reinforce each other, yielding a greater positive impact on farmers′ marketing performance than either channel used in isolation [40]. This empirical review synthesizes findings from developing countries and Ethiopia in particular to highlight how key demographic, socioeconomic, and institutional factors jointly drive or hinder this essential communication synergy in vegetable marketing.

With respect to age, empirical findings consistently indicate a negative and statistically significant relationship with ICT utilization for accessing market information [41]. Older farmers often face greater difficulty adapting to ICT tools because of lower digital literacy, visual or physical limitations, higher risk aversion, and cost constraints [42, 43]. Consequently, older vegetable growers rely more heavily on indigenous, face‐to‐face communication channels, such as local village markets and trusted friends networks, where they leverage extensive experiential knowledge of local trade dynamics and established social capital [44]. Multiple studies across developing agricultural contexts reaffirm that younger farmers are significantly more likely to adopt mobile phones and digital platforms for agricultural marketing than their older counterparts, who face substantial structural constraints in ICT tool usage [45, 46]. However, this age disparity does not necessarily preclude older farmers from benefiting from ICT market insights. Rather, it fosters a critical intergenerational synergy: younger, more ICT‐proficient household or community members serve as digital intermediaries.

Gender disparities in access to and control over ICTs remain a significant challenge across developing countries. Empirical evidence from smallholder agricultural systems in Ethiopia including the Oromia region demonstrates that female‐headed households and women farmers face systematic barriers to adopting ICT‐based market tools [47]. Lower digital literacy, constrained intrahousehold control over cash assets, restricted spatial mobility because of reproductive and domestic burdens, and unequal access to formal agricultural extension services significantly limit women′s independent ownership and direct usage of mobile tools for market tracking [48]. Consequently, quantitative model results frequently reveal a negative and statistically significant association between female gender and direct ICT utilization for market information. However, within agrarian Oromo communities, women play a crucial role in postharvest handling, localized retail marketing, and informal community networks. Women serve as central nodes in ICCs, such as microlevel neighborhood discussions, Iddirs, informal labor‐sharing groups (Dabo/Jiggi), and local weekly markets where high‐trust market intelligence and price observations are routinely exchanged. Recognizing this contextual dualism, in which women are structurally constrained in direct ICT adoption yet highly active in traditional information networks, is critical for designing blended communication strategies that leverage women′s central social role to facilitate ICT–ICC communication synergies.

Education level is consistently identified across empirical literature as one of the most powerful and positive determinants of ICT utilization in smallholder agriculture, showing statistically significant positive effects in binary logit and probit model estimations. Rather than merely acting as an indicator of literacy, educational attainment serves as the cognitive foundation for communication synergy. Higher education enhances a farmer′s information‐processing capacity specifically the ability to decode, evaluate, and reconcile abstract, quantitative data delivered via ICTs (such as distant market price feeds, trend forecasts, and standardized weight metrics) with the qualitative, context‐rich knowledge transmitted through ICCs, such as peer market observations and local trader reputation [49].

This cognitive reconciliation provides a critical micromechanism for social learning theory (SLT). According to SLT, individuals acquire and adopt new behaviors not only through direct experience but also by observing, interpreting, and modeling information within their social environment. Educated farmers function as key analytical nodes within this social framework: They process complex digital market signals, validate them against local indigenous observations, and model informed marketing decisions (such as optimal timing or spatial arbitrage). Other community members then observe these outcomes and adopt the blended information through trusted, informal social networks. Thus, individual educational attainment not only drives personal digital adoption but also catalyzes community‐wide communication synergy by turning raw ICT data into actionable, socially reinforced local knowledge.

Farm and nonfarm income is consistently identified as a statistically significant positive determinant of communication strategy choice. Empirical studies show that higher income increases a farmer′s capacity to afford the fixed costs of ICTs (devices and maintenance) and the variable costs (airtime and data bundles) necessary for continuous information flow [49]. This positive financial effect acts as a foundational prerequisite for synergy, as the successful combination of channels is dependent on the farmer′s ability to maintain the ICT component, thereby avoiding complete reliance on free but slower ICCs. Inconsistent results were also found by [41], who revealed that farm income is negative and significantly associated with the use of ICTs for accessing agricultural marketing information. Individuals with higher incomes might have access to alternative, perhaps more traditional, or personalized sources of market information that they find more reliable or convenient, such as hiring market consultants, using premium services, or relying on established business networks. These alternatives reduce their reliance on ICT to access such information. Similarly, higher income individuals might not perceive ICT as necessary for their market information needs, primarily if they are engaged in larger scale operations where direct or traditional methods are preferred over ICT solutions.

There was a positive and significant correlation between access to credit and the use of the ICT tools. Empirical studies show that access to credit has the potential to increase the financial ability of smallholder farmers to use ICT tools in agriculture [50, 51]. The smallholder farmers may access credit from formal and informal institutions such as banks, farmer groups, friends, and relatives [52]. Similarly, the study made by [53] found that access to agricultural credit could be among other factors that significantly facilitated the use of ICT tools in communicating agricultural extension information. Indigenous channels are crucial for trust and literacy: Indigenous communication systems (such as interpersonal networks, community leaders, and town criers) are essential for building trust, overcoming literacy barriers, and effectively disseminating information about financial services and ICT use to marginalized populations. Indigenous channels like friends, relatives, neighbors, extension agents, and community gatherings are often among the most preferred sources of information for farmers.

Farm size has positive and significant effect on ICT use [54]. This is due to the fact that farmers with higher farm sizes tend to seek higher information for the betterment of agriculture rather than their counterparts with less landholding [55]. Other findings do not align with the above findings revealing that farm size has a negative but significant effect on using ICT to access market information [56]. This means that as the size of the farm increases, the likelihood of using ICT to access market information decreases. This could be because larger farms already have established market channels and relationships with buyers, reducing the need to seek out market information via ICT actively. These farms might rely on face‐to‐face or long‐term contracts instead of using ICT to find new markets or price information.

Market distance demonstrates a positive influence on synergy; the greater the distance, the higher the need to rely on the time‐saving efficiency of ICTs, which then necessitates robust validation through local ICCs [57]. In contrast, Mutuma et al. [58] found that a shorter distance to the market is potentially an incentive for vegetable farmers′ usage of ICT communication channels for marketing vegetable crops.

Furthermore, membership in formal or informal social organizations demonstrates a positive and powerful, consistent positive influence on communication synergy. In this sense, the organizational structure (ICC) serves to maximize the reach and impact of the digital message (ICT), yielding a synergistic effect that is highly valued by farmers. This factor is central to the synergy definition: ICC provides the collective action platform where the individual market alert (ICT) is validated, acted upon, and converted into collective market power, leading to measurable economic benefits [59]. Likewise, extension contact exerts a positive influence, as extension agents are key synergy facilitators, integrating ICT tools into conventional group training and thereby validating the credibility of the technological channel [59].

In terms of infrastructure, network availability (mobile/internet) and electric power availability are highly consistent in their function as nonnegotiable positive prerequisites. Network access played a crucial role in ICT usage, with a significant positive effect [60]. Specifically, access to electricity is a significant factor in the use of ICT for agricultural services in Ethiopia [60]. Similarly, the study of Abdurrauf et al. [61] highlighted that access to electricity is critical for the effective use of mobile phones for agricultural services, as their functionality depends heavily on reliable electricity. The presence of these factors, therefore, enables the operational viability of ICTs, which is the essential first step toward blending digital and traditional communication streams.

### 2.6. Integrated Theoretical Framework Paragraph (Diffusion of Innovations [DOI] and SLT Synergy)

Although DOI [62] and SLT [63] are frequently applied independently in communication studies, their integration provides a complementary framework for explaining synergistic channel use in smallholder vegetable marketing. DOI accounts for the individual, structural decision‐making process through which a farmer evaluates and adopts ICT tools based on attributes, such as perceived relative advantage (e.g., accessing distant wholesale price data) and compatibility with existing farming routines. However, DOI alone struggles to explain why smallholders, even after acquiring ICT tools, continue to rely heavily on informal local networks. This is where SLT provides the essential social mechanism: It demonstrates how observational learning, peer modeling, and social validation within ICCs, such as Iddirs, local market exchanges, and trusted trader networks (delalas), reduce the perceived risk and complexity of new digital market signals.

In the highly uncertain and time‐sensitive context of perishable vegetable marketing in rural Ethiopia, these two theories operate in a continuous feedback loop. Digital tools (DOI lens) deliver rapid, macrolevel market intelligence, whereas indigenous networks (SLT lens) serve as the social apparatus where smallholders crossvalidate, interpret, and observe the real‐world trading outcomes of early adopters before taking market action. Consequently, individual technological adoption (DOI) and collective social learning (SLT) do not function as competing pathways but as mutually reinforcing pillars that drive concurrent and synergistic communication strategies among rural farmers. The conceptual framework of the study is illustrated in Figure 1.

### 2.7. Conceptual Framework of the Study

**Figure 1:**
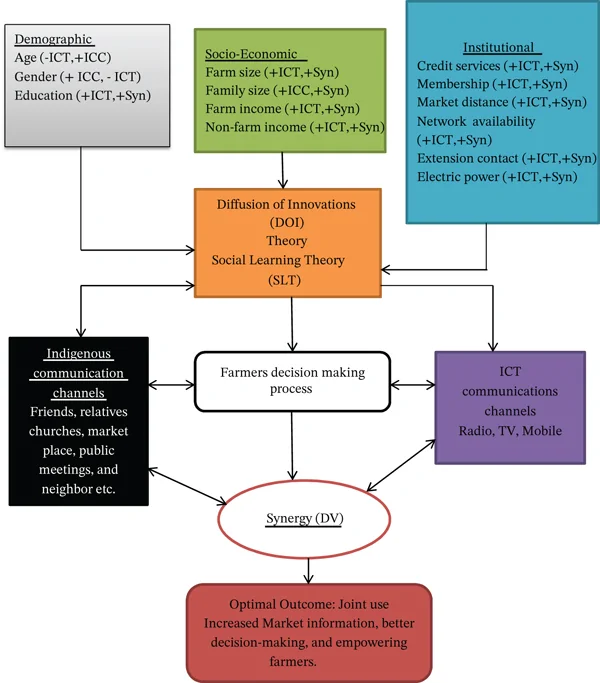
Conceptual framework of the study. *Source:* Author′s design (2024).

## 3. Methodology

### 3.1. Description of the Study Area

The study was conducted in the Haramaya and Kombolcha Districts of the East Hararghe Zone, Ethiopia. Kombolcha Woreda is situated 17 km north of Harar and 542 km east of Addis Ababa. Its altitude ranges from 1200 to 2460 m above sea level, spanning agroclimatic zones from *Woina Dega* (midaltitude) to *Kola* (lowland). Annual rainfall varies between 600 mm and 900 mm with a bimodal, erratic pattern, whereas mean temperatures range from 16°C to 25°C. The district has a population of approximately 195,870, with over 90% residing in rural areas. Major crops include potatoes, cabbage, onions, carrots, sorghum, maize, and khat. Notably, Kombolcha is a significant national hub for potato trading [64].

Haramaya District is located 12 km west of Harar and 524 km east of Addis Ababa. Its altitude ranges from 1214 to 2066 m above sea level, also spanning Woina Dega and Kola zones. It borders the Dire Dawa Administrative Council, Kombolcha, Fedis, Kurfa Chele, and Kersa Districts. Haramaya covers a total area of approximately 521.63 km^2^. Rainfall and temperature patterns are similar to Kombolcha. Farming includes both rain‐fed and irrigation methods, with major production focusing on sorghum, maize, pulses, and high‐value vegetables like potatoes, onions, and lettuce, alongside khat for domestic and export markets [64]. Empirical survey data across East Hararghe indicates basic mobile phone ownership rates among rural smallholder households estimated around 67.7%. However, field assessments reveal that over 85%–90.6% of active users utilize phones strictly for basic voice calls to local contacts, whereas advanced agricultural m‐services, SMS updates, and internet‐based tools remain under 20%. Rural smartphone ownership in the region remains low (< 12%–15%) because of erratic 3G/4G coverage in rural kebeles and low grid electricity access (< 35%), forcing reliance on off‐grid solar or paid rural charging shops and usage of generator power pump water for irrigation [65, 66]. Map of the East Hararghe Zone and the study area are illustrated in Figure 2.

**Figure 2:**
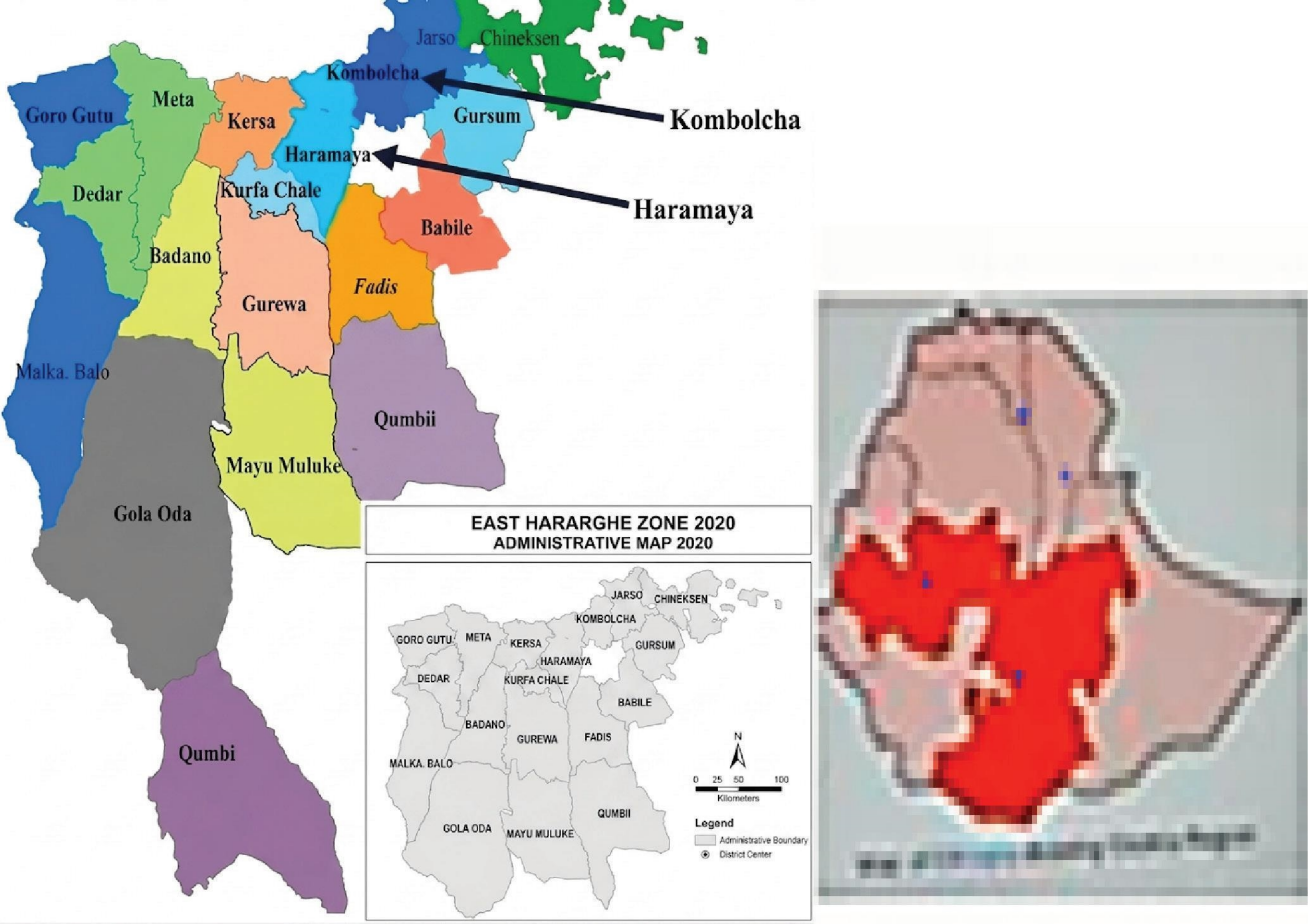
Map of the study area and East Harerghe Zone. *Source:* Ethio – GIS, 2020 EC.

### 3.2. Population and Sampling Procedure

The study focused on smallholder farmers in the Haramaya and Kombolcha Districts. The target populations were smallholder farmers from four kebeles (villages) in Haramaya Woreda and three kebeles in Kombolcha Woreda.

In this study, probability and nonprobability sampling techniques were applied. A multistage sampling technique was employed to select the study sites and determine the sample size. First, Haramaya and Kombolcha Districts were purposively selected based on their potential as leading vegetable‐producing areas in the East Hararghe Zone. Within each woreda, kebeles were identified based on vegetable production. In the second stage from the total of 33 kebele in Haramaya district, 17 kebele were selected purposively because 17 kebele are known as vegetable growers in the Haramaya district. The same procedures were applied in Kombolcha from the total 19 kebele found in Kombolcha District, 11 kebele were selected purposively because of the 11 kebeles known as vegetable growers in the Kombolcha District. In the third stage, a stratified random sampling approach was used to select the kebeles. Vegetable‐producing kebeles in each district were stratified into distinct homogenous groups based on a combination of four criteria: annual vegetable production volume, average landholding size, proximity to major market center, and population size in their order of importance from first to fourth, respectively. In Haramaya District, the 17 vegetable‐growing kebeles were stratified into four distinct groups. From each of these four strata, one kebele was randomly selected using a lottery method, ensuring that all variations of vegetable‐farming contexts were represented in the final selection of four kebeles (Kuro, Damota, Xiniqe, and Tuji Gabisa). Similarly, in Kombolcha District, the 11 vegetable‐growing kebeles were stratified into three distinct groups based on the same criteria. One kebele was randomly selected from each of the three strata, yielding three kebeles (Bilisuma, Qeyrensa, and Burqa Negaya). Thus, a total of seven kebeles were selected for the study. The total number of HHs in the selected seven kebeles in Haramaya and Kombolcha Districts was 10,063 HHs.

### 3.3. Sample Size and Selection

The sample size for collecting quantitative data for this research was determined using Yamane′s [67] formula for finite populations:

(1) n=N1+Ne2,

*where* *n* designates the sample size the research uses, *N* designates the population size (10,063), and *e* designates the level of precision (*e* = 0.05 at 95% confidence level).

A 95% confidence level (*α* = 0.05) was selected to ensure that the sample statistics reliably reflect the true population parameters within a standard error bound, minimizing the risk of Type I errors (false positives). In social science and rural development research, a 95% confidence level represents the standard benchmark for statistical significance, striking an optimal balance between minimizing sampling bias and maintaining practical field feasibility. Furthermore, a 5% precision level (*e* = 0.05) was selected because it balances statistical accuracy with logistical and financial feasibility, rendering 385 respondents a robust and representative sample for empirical analysis.

Based on this formula, a minimum sample size of 385 smallholder vegetable farmers was determined. To ensure fair representation across the study locations, a multistage sampling procedure was employed. First, the total sample of 385 was distributed between the study areas using a probability proportional to size (PPS) sampling technique, resulting in 199 farmers from Haramaya and 186 from Kombolcha. Finally, to select the specific households within each rural *kebele*, systematic random sampling was applied as described by [68]. An element of randomness was introduced by using a random number generator to select the starting household unit. From that random starting point, households were systematically selected using a calculated sampling interval (*k*) until the required sample quota for each kebele was secured.

Seven focus group discussions (FGDs) (one per kebele) were conducted, each consisting of seven members (34 males and 15 females total) selected purposively based on their agricultural experience. These sessions lasted 35–50 min and used semistructured checklists to explore perceptions of indigenous versus modern communication channels. Additionally, 25 key informant interviews (KIIs) were conducted with 18 farmers, four extension agents, and three agricultural officials to provide institutional context. Secondary data were gathered from published articles, CSA documents, and district agricultural reports.

### 3.4. Data Types, Sources, and Collection Methods

This study employed a mixed‐methods approach, utilizing both qualitative and quantitative data from primary and secondary sources. Primary data were collected between June and September 2023 using structured interview schedules, FGDs, and KIIs. The structured questionnaire was pretested with 30 nonsample households in Kuro Kebele to ensure clarity, validity, and reliability [69].

To ensure methodological rigor in analyzing the qualitative data from FGDs and KIIs, a structured thematic analysis approach was implemented. Audio recordings and field notes were first transcribed verbatim and translated into English. Initial thematic codes were independently developed and assigned to the transcripts by two researchers. To establish interrater reliability, the coded segments were compared, and any discrepancies in thematic categorization or code assignments were discussed and resolved through joint review until a consensus was reached (achieving an intercoder agreement above 85%). The finalized thematic categories were then synthesized and integrated with the quantitative findings to triangulate the results.

### 3.5. Data Processing and Analysis

Quantitative data were analyzed using descriptive statistics (mean, standard deviation, frequency, and percentages) alongside inferential econometrics via SPSS Version 27 and STATA 15.0. Qualitative data were analyzed using thematic interpretation to validate, contextualize, and elaborate on the quantitative findings.

To identify the factors influencing the simultaneous use of indigenous and ICT‐based communication channels, a bivariate probit model (BVPM) was employed. Although standard probit models analyze a single binary choice in isolation, smallholder farmers often do not select communication channels independently; instead, they mix and match available traditional and modern information sources. The BVPM is ideal because it models the joint determination of two correlated binary choices simultaneously. Analyzing these decisions using two separate, independent univariate probit models would assume that the choice to use indigenous channels has no statistical connection to the choice to use ICTs. If an unobserved relationship exists, separate models ignore this interdependence, leading to inefficient and biased parameter estimates. By contrast, the BVPM explicitly models this joint reliance, capturing the synergy, complementarity, or substitution between the two channel types [70].

The econometric specification of the BVPM is framed through two underlying latent variable equations:

(2) Y1i∗=β1X1 i+ϵ1i,

(3) Y2i∗=β2X2i+ϵ2i,

where the observed binary choices are expressed as

(4) Y1i∗=100 if Y1i∗> farmers use indigenous channels,otherwise .

(5) Y2i∗=100 if Y2i∗> farmers use ICT based channels,otherwise .

#### 3.5.1. Contextual Translation of Model Components

1. Latent variables $\left(Y_{1i}^{*}\;\text{and}\;Y_{2i}^{*}\right)$ represent farmer *i*′s unobservable propensity, underlying desire, or perceived utility gained from utilizing ICCs ($Y_{1i}^{*}$) and ICT‐based tools ($Y_{2i}^{*}$), respectively. Although researcher observation is restricted to the binary outcome whether the farmer actually uses the channel (*Y* = 1) or not (*Y* = 0), the latent variable reflects the continuous propensity driving that decision.
2. Explanatory variables ($Y_{1i}^{*}\;\text{and}\;Y_{2i}^{*}$) represent vectors of socioeconomic, institutional, and demographic covariates (e.g., age, education level, farming experience, extension contact, market distance, off‐farm income, and radio/mobile phone ownership) that influence information channel selection.
3. Error terms (**ϵ**_1**i**,_ **ϵ**_2_ **i**) and correlation coefficient (**ρ**): The error terms ($Y_{1i}^{*}\;\text{and}\;Y_{2i}^{*}$) capture unobserved farmer‐level characteristics, institutional factors, or environmental variables that affect channel selection but are not directly measured in the survey, such as individual risk tolerance, innate digital literacy, trust in local social networks, or general enthusiasm for market information.

The correlation parameter ($\rho =\text{Cov}\;\left.\left[Y_{1i}^{*}\;\text{and}\;Y_{2i}^{*}\right)\right]$) measures the degree of interdependence between these unobserved factors across both choices:

• Positive correlation (*ρ* > 0 and statistically significant) indicates synergy or complementarity. Substantively, this means that unobserved factors that push a farmer to seek vegetable marketing information through indigenous channels (e.g., high social capital or strong community participation) also make that same farmer more inclined to utilize ICT tools.
• Negative correlation (*ρ* < 0 and statistically significant) indicates substitution. Substantively, unobserved factors driving a farmer toward traditional channels lower their likelihood of adopting ICT platforms.
• Insignificant correlation (*ρ* = 0) indicates that decisions are statistically independent, confirming that two separate univariate probit models would have been sufficient.
• Joint probability outcome: The joint probability that a smallholder farmer simultaneously utilizes both channel types is modeled using the bivariate standard normal cumulative distribution function (*Φ*_2_):

PY1i∗=11,Y2i∗==Φ2β1X1 i,β2X2iρ .

This bivariate normal distribution calculates the joint probability by accounting for both observed socioeconomic predictors (*X*_*i*_) and the underlying correlation (*ρ*) between unobserved information‐seeking behaviors.

## 4. Results and Discussion

### 4.1. Description of Demographic, Socioeconomic, and Institutional Variables

Demographic, socioeconomic, and institutional characteristics access patterns play a definitive role in shaping how smallholder vegetable farmers interact with agricultural information. These factors determine not only a household′s resource capacity to adopt ICT technologies but also their reliance on traditional, localized knowledge systems.

#### 4.1.1. Continuous Household Characteristics

The baseline continuous characteristics of the sampled farm households reveal a resource‐constrained, mature farming profile with varying capacities to absorb new information systems (Table 1). As shown in Table 1, the mean age of household heads was 41.84 years, reflecting a middle‐aged, economically active farming population. The average educational attainment was 4.75 years, indicating that most respondents had completed lower primary schooling.

A critical finding emerges from the structural imbalance between land ownership and demographic pressure. The mean farm size is exceptionally small at 0.56 ha (ranging from 0.25 to 2.00 ha), whereas the average family size is 6.08 persons (ranging from 2 to 15 members). This yields an average land‐to‐person ratio of less than 0.10 ha per individual, underscoring intense land scarcity, fragmented holdings, and high household reliance on high‐value, small‐scale vegetable commercialization to maintain rural livelihoods.

The mean annual farm income across the sample was ETB 150,477.40 (SD = 91,165.33). To provide international context for readers outside Ethiopia, at the time of data collection, this average represented approximately 2735 USD (or 450 USD per capita annually across an average household of six members, yielding roughly 1.23 USD per person per day).

• Poverty threshold alignment: Compared with national and international poverty benchmarks, households at the lower income extreme (ETB 20,496.00/year or 0.17 USD per capita/day) fall severely below the World Bank′s extreme poverty line (2.15 USD per person/day). Conversely, households at the upper extreme (ETB 478,500.00/year or 3.90 USD per capita/day) sit comfortably above international thresholds.
• Income inequality: The wide spread between minimum (ETB 20,496) and maximum (ETB 478,500) earnings reflects substantial economic inequality within smallholder vegetable producers. This dispersion is primarily driven by disparities in access to year‐round irrigation, proximity to major high‐road market hubs, land quality, and adoption of intensive input practices. Households in the top income quartile leverage commercialized vegetable cropping to achieve relative wealth, whereas resource‐poor smallholders in the lower quartiles remain in subsistence traps, directly impacting their capacity to afford ICT tools or access fee–based market information.

Finally, market distance—the physical infrastructure environment—presents a notable barrier: Farmers walk an average of 5.83 km to reach the nearest urban market center, with some traveling up to 9 km, reinforcing the importance of localized or remote communication alternatives to bridge the physical distance.

**Table 1:** Descriptive statistical results for sample households′ continuous variables (*n* = 385).

Variables	Mean	Std. deviation	Min	Max
Age	41.84	9.186	25	67
Education	4.75	1.637	1	10
Farm size	0.5609	0.29392	0.25	2.00
Family size	6.08	2.495	2	15
Farm income	150,477.4	91,165.33217	20,496.00	478,500.00
Market distance	5.83	2.461	2	9

*Note: Source(s):* SPSS output (2024).

#### 4.1.2. Categorical and Institutional Characteristics

Institutional variables, social connectivity, and basic infrastructure dictate the reach and effectiveness of different communication pathways. As shown in Table 2, the sample is predominantly male‐headed, with 85.5% male respondents (*n* = 329) and 14.5% female respondents (*n* = 56). This gender distribution was incidental rather than intended, as the sampling frame relied on registered head‐of‐household lists maintained at local kebele administrative offices. In rural eastern Ethiopia, land registration and primary farming decision‐making responsibilities are culturally and socioeconomically designated to male household heads, resulting in a lower representation of independent female‐headed households.

Although this distribution accurately reflects the demographic structure of landholding decision‐makers in the target kebeles, it presents a methodological limitation regarding the generalizability of gender‐related findings. Institutional barriers such as reduced access to formal extension services, lower resource ownership, and restricted access to public ICT infrastructure frequently affect female farmers differently. Consequently, although the econometric estimates reflect household‐level decisions driven primarily by male heads, caution must be exercised when generalizing these findings to women farmers across the broader agricultural sector.

The community exhibits low economic diversification; a vast majority (83.9%) rely strictly on farm income, with only 16.1% engaging in nonfarm activities.

Institutional support shows mixed availability. Although a strong majority accessed credit (68.8%) overwhelmingly from informal rural networks and 61.0% maintained memberships in local organizations, these community structures provide prime environments for the natural flow of indigenous, word‐of‐mouth information.

In terms of communication readiness, the data reveals high baseline connectivity alongside severe infrastructural bottlenecks. Encouragingly, 74.5% of farmers report active access to market information, 80.0% live within mobile network coverage zones, and 74.3% maintain steady contact with public agricultural extension agents. These high rates signify a strong foundation for scaling ICT agricultural interventions.

However, a massive structural constraint remains: 68.6% of the households completely lack electric power at home. Even if a farmer has network coverage and a mobile phone, the absence of household electricity complicates device charging, creating a unique operational barrier that can disrupt the consistency of ICT channel usage and reinforce a reliance on indigenous, face‐to‐face communication systems. This coincides with the World Bank and Ethiopian Electric Utility estimates (2020–2023); national rural electrification access in Ethiopia sits at approximately 30%–35%, compared with over 90% in urban centers. In the study area, the lack of domestic electricity creates a practical operational barrier. Even when a farmer possesses a mobile phone and resides within cellular network coverage, charging handset batteries requires traveling to market towns or paying commercial charging kiosks. This logistical friction regularly interrupts mobile phone usage, disrupting the continuity of digital information channels and forcing smallholders to continuously rely on indigenous, face‐to‐face communication systems as a dependable backup.

**Table 2:** Descriptive statistical results for sample households′ categorical variables (*n* = 385).

Variables	Category	Number	Percent
Gender	Male	329	85.5
	Female	56	14.5
Nonfarm income	No	323	83.9
	Yes	62	16.1
Credit	No	120	31.2
	Yes	265	68.8
Membership	No	150	39.0
	Yes	235	61.0
Access to market info	No	98	25.5
	Yes	287	74.5
Mobile network	No	77	20.0
	Yes	308	80.0
Extension contact	No	99	25.7
	Yes	286	74.3
Electric power	No	264	68.6
	Yes	121	31.4

*Note: Source(s):* SPSS output (2024).

### 4.2. Determinants of the Joint Utilization of Indigenous and ICT Communication Channels: A Bivariate Probit Analysis

#### 4.2.1. Model Diagnostics and Interdependence Validation

The dependent variables of this study represented the use of two distinct communication pathways for sharing vegetable crop marketing information: indigenous channel use (coded as 1 if the farmer used ICCs and 0 if otherwise) and ICT channel use (coded as 1 if the farmer used at least one ICT tool, such as radio, television, or mobile phone and 0 if otherwise). Both types of communication use were modeled simultaneously to capture their potential interdependence. To identify the factors influencing these choices, this study employed a BVPM, which is suited for analyzing dual, binary dependent variables with correlated error terms. Rather than running separate, isolated probit models, the BVPM estimates both equations jointly.

The model evaluated 14 independent variables, consisting of six continuous and eight categorical variables, hypothesized to influence the joint use of these channels. These explanatory factors included demographic variables (age, sex, and education), socioeconomic variables (family size, access to credit, farm income, nonfarm income, and farm size), and institutional or infrastructural variables (access to market information, distance to market, membership, extension contact, mobile network coverage, and electrical power supply). All statistical analyses were executed using SPSS Version 27 and STATA 15.0 software packages.

Prior to executing the BVPM, diagnostic tests were conducted to evaluate potential multicollinearity among the explanatory variables. The variance inflation factor (VIF) and tolerance levels were examined using linear regression diagnostics. Variables with a VIF value of 5.0 or above reflect moderate‐to‐high multicollinearity, whereas values of 10.0 or above signal severe multicollinearity that threatens model stability [71].

The initial diagnostic assessment identified an elevated VIF value (VIF = 5.03) for the variable “access to market information,” driven by its strong correlation with primary institutional variables, particularly extension contact and mobile network coverage. To preserve model stability and prevent inflated standard errors, access to market information was excluded from the final econometric model. Following this exclusion, multicollinearity diagnostics were rerun across the remaining 13 explanatory variables (consisting of six continuous and seven categorical variables). All remaining 13 variables exhibited VIF values well below the conservative threshold of 5.0 (with a mean VIF of 1.42, ranging from 1.08 to 2.15), confirming that severe multicollinearity was successfully eliminated and confirming the structural integrity of the specification.

To assess whether excluding access to market information introduced omitted variable bias, sensitivity checks and theoretical evaluations were conducted. Substantively, market information access in the study area is not an isolated exogenous driver; rather, it is directly mediated through underlying institutional mechanisms, namely, direct extension contact, mobile network availability, credit access, and active membership in local organizations (Iddirs and cooperatives). Because these primary structural channels remain fully specified within the model, they capture the practical pathways through which market information is accessed and utilized. Consequently, the exclusion of access to market information did not distort or alter the directional signs, statistical significance, or substantive interpretation of the remaining coefficients, ensuring that the model estimates remain consistent and unbiased.

The analysis results of the BVPM are given in Table 3. The likelihood ratio test for the correlation coefficient (rho) evaluates whether the unobserved factors (residuals) influencing the two equations are correlated. The estimated correlation coefficient (rho) was found to be positive and statistically significant (rho = 0.498, *p* < 0.05). This statistically significant positive rho confirms that unobserved factors influencing the adoption of ICCs are positively associated with unobserved factors driving ICT channel adoption, validating the joint estimation approach over two separate univariate probit models.

Although a positive and significant rho indicates strong statistical interdependence and a tendency for farmers to adopt both channels jointly, it reflects shared unobserved household or contextual characteristics rather than direct empirical proof of functional synergy or complementarity. The overall model diagnostics further demonstrate robust fit, with an overall Wald chi‐square statistic of 94.661 (*p* < 0.001), confirming that the explanatory variables jointly exert a statistically significant effect on farmers′ communication choices.

#### 4.2.2. Factors Influencing the Joint Adoption of Indigenous and ICT Communication Channels

As shown in the bivariate probit estimation (Table 3), the age of the household head acts as a distinct bifurcating factor, positively driving reliance on ICCs while negatively influencing ICT adoption. The findings show that the age of the household farmer had a positive coefficient of 0.014 and was marginally significant at the 10% level (*p* < 0.10). Holding other variables constant, a 1‐year increase in age increases the probability of using ICCs by 0.014. This implies that older smallholder farmers rely more on indigenous channels such as respected elders, friends, neighbors, relatives, and farm visits because their extensive experience helps them select trusted sources for their information needs. This result aligns with [72], who noted that older household heads often prefer indigenous over exogenous channels. Conversely, age negatively influenced ICT channel usage with a coefficient of −0.023, significant at the 1% level (*p* < 0.01). Each additional year of age reduces the probability of using ICT channels for vegetable marketing by 0.023. Unlike younger farmers who actively leverage ICT for market, production, and technical information, older farmers are often less familiar with these technologies and more hesitant to adopt them, a finding consistent with [73, 74]. Furthermore, this aligns with our theoretical framework: Under DOI theory, perceived complexity reduces ease of use, whereas under SLT, lower self‐efficacy and fewer observational opportunities hinder technology adoption.

This generational divide is clearly illustrated in the descriptive crosstabulation (Table 4). Farmers who rely exclusively on indigenous channels exhibit the highest mean age (48.62 years), whereas those who rely exclusively on ICT channels are substantially younger, with a mean age of 35.18 years, a striking generational gap of over 13 years. Interestingly, farmers who practice dual‐channel usage (joint adoption group) occupy an intermediate middle‐aged demographic (mean age of 40.85 years), demonstrating that middle‐aged farmers are uniquely positioned to bridge traditional social networks with modern digital technologies.

Education had a positive but statistically insignificant effect on indigenous channel usage (*β* = 0.009, *p* > 0.10). This implies that formal schooling does not diminish a farmer′s reliance on indigenous communication methods, underscoring their enduring cultural and social importance within the community. Conversely, education positively and marginally significantly influenced ICT channel usage (*β* = 0.086, *p* < 0.10). Holding all other variables constant, each additional grade completed increases the probability of using ICT tools for agricultural communication by 0.086. Formal education equips farmers with the basic literacy and numeracy required to operate tools like mobile phones and radios while enhancing their ability to evaluate market information and make informed decisions for vegetable marketing. Education thus acts as a bridge, enabling smallholder farmers to integrate modern technology with existing knowledge practices. This outcome aligns with DOI theory, which asserts that higher education enhances a farmer′s ability to recognize the relative advantages of an innovation while reducing its perceived complexity, supporting the findings by [75].

Farm size had a positive but statistically insignificant effect on indigenous channel usage (*β* = 0.106, *p* > 0.10), indicating that landholding size does not dictate reliance on traditional communication methods. Conversely, farm size exhibited a highly significant positive effect on ICT usage (*β* = 0.361, *p* < 0.01). Holding all other variables constant, a 1‐ha increase in farm size increases the probability of utilizing ICT tools by 0.361. Farmers with larger landholdings have both the economic capacity and a greater operational incentive to adopt technology. Managing larger scale production requires frequent, detailed market information to optimize logistics and profitability. Theoretically, this finding reflects the “relative advantage” concept within DOI theory, as the financial returns of larger farms heighten the perceived value of ICT investments. From an SLT perspective, larger scale farmers often serve as early adopters or community leaders whose visible, successful integration of ICTs provides a model for observational learning among peers, aligning with [75].

Similarly, farm income showed a positive but statistically insignificant relationship with indigenous communication channel usage (*β* = 0.096, *p* > 0.10), implying that income level does not dictate traditional method usage. In contrast, farm income had a statistically significant positive effect on ICT usage (*β* = 0.035, *p* < 0.05). Higher household income increased the likelihood of ICT communication channel usage by 0.035, holding other variables constant. Wealthier farmers possess the financial means to purchase devices (mobile phones, radios, and televisions) and cover ongoing airtime and data costs. This supports the financial dimension of DOI′s relative advantage and enhances farmer self‐efficacy under SLT. Furthermore, higher income farmers benefit from the complementary interaction between word‐of‐mouth indigenous networks and real‐time ICT tools for market information exchange. These findings concur with [76, 77] and [78, 79].

**Table 3:** Bivariate probit model estimation on indigenous channels and ICTs channels.

Variables	Indigenous communication channels use			ICTs communication channels use		
	*β*	SE	*p*	*β*	SE	*p*
Age	0.014 ^∗^	0.008	0.076	−0.023 ^∗∗∗^	0.008	0.004
Gender	−0.031	0.207	0.881	0.047	0.208	0.822
Education	0.009	0.049	0.851	0.086 ^∗^	0.05	0.084
Farm size	0.106	0.242	0.66	0.361 ^∗∗∗^	0.255	0.001
Family size	0.048	0.032	0.137	0.043	0.033	0.192
Income	0.096	0.043	0.942	0.035 ^∗∗^	0.034	0.025
Nonfarm income	−0.015	0.196	0.94	−0.151	0.196	0.441
Credit	0.963 ^∗∗∗^	0.167	0.001	1.096 ^∗∗∗^	0.171	0.001
Membership	0.029	0.148	0.848	0.108	0.151	0.473
Market distance	0.018	0.033	0.58	−0.005 ^∗∗^	0.034	0.005
Mobile network	0.873 ^∗∗∗^	0.204	0.001	0.888 ^∗∗∗^	0.212	0.001
Extension contact	0.182 ^∗∗^	0.201	0.035	0.152 ^∗∗^	0.206	0.029
Power supply	−0.369 ^∗∗^	0.161	0.022	0.541 ^∗∗∗^	0.162	0.001
Constant	−0.457 ^∗∗∗^	0.537	0.014	−0.623 ^∗∗∗^	0.546	0.002
athrho	0.498 ^∗∗^	0.067	0.018			
Mean dependent variable		0.588	SD dependent variable		0.464	
Number of observation		385	Chi‐square		94.661	
Prob > chi^2^		0.001				

*Note: Source:* Survey result (2023).  ^∗^,  ^∗∗^, and  ^∗∗∗^ mean significant at 10%, 5%, and 1%, respectively.

**Table 4:** Crosstabulation of mean age across communication channel usage profiles (*n* = 385).

Channel usage profile	Sample (*n*)	Share (%)	Mean age (years)	Std. deviation
Indigenous channels only	98	25.5	48.62	7.84
ICT channels only	64	16.6	35.18	6.21
Dual‐channel users (synergy group)	162	42.1	40.85	8.12
Nonusers	61	15.8	43.40	9.75
Total sample	385	100.0	41.84	9.19

*Note: Source:* Survey results (2023/2024).

The availability of access to credit services exerted a strong positive and highly significant influence on the propensity to utilize both information streams, yielding coefficients of *β* = 0.963 (*p* < 0.01) for ICCs and *β* = 1.096 (*p* < 0.01) for modern ICT channels (Table 3). These results indicate that smallholder households with access to credit are significantly more likely to engage with both indigenous interpersonal networks and ICT tools than their noncredited counterparts. Credit acts as a financial catalyst that empowers farmers to leverage both information streams simultaneously. Credit alleviates capital constraints, enabling farmers to purchase ICT hardware and operational services (airtime and data) while also funding participation costs associated with indigenous channels (transport to market centers and community meetings). This dual enhancement strongly supports our conceptual integration of DOI and SLT, concurring with [80].

With regard to market distance, geographic distance to market centers had a positive but statistically insignificant effect on indigenous communication channel usage (*β* = 0.018, *p* > 0.10). In contrast, market distance had a statistically significant negative effect on ICT channel usage (*β* = −0.005, *p* < 0.01). Holding all other variables constant, each 1‐km decrease in distance to the market center increases the likelihood of ICT channel usage by 0.005. Farmers located closer to markets are slightly more likely to adopt ICT tools because frequent proximity to market nodes, output dealers, and visiting peers provides greater exposure to market extension services and digital information channels, aligning with [81].

Mobile network availability had a strong positive and highly significant effect on both indigenous channel usage (*β* = 0.873, *p* < 0.001) and ICT channel usage (*β* = 0.888, *p* < 0.001), as detailed in Table 3. Farmers with network access exhibited a substantially higher likelihood of utilizing both traditional and modern communication channels compared with those in uncovered areas. The positive influence on indigenous channels reveals a key complementary relationship: Mobile networks do not replace traditional interpersonal communication but rather amplify its geographic reach and speed by enabling direct calls to peers, relatives, and market leaders [82].

Contact with extension agents exerted a statistically significant positive influence on both indigenous (*β* = 0.182, *p* < 0.05) and ICT communication channels (*β* = 0.152, *p* < 0.05). Each additional contact with an extension worker increased the probability of utilizing indigenous and ICT channels by 0.182 and 0.152, respectively. Extension agents function as “change agents” under DOI who introduce innovations, and as role models under SLT who demonstrate multichannel usage, consistent with [82].

As shown in Table 3, access to a reliable electric power supply had a statistically significant negative effect on indigenous communication channel usage (*β* = −0.369, *p* < 0.05) while exerting a positive and highly significant effect on ICT channel usage (*β* = 0.541, *p* < 0.01). Substantively, access to household electricity does not imply that farmers completely abandon traditional, face‐to‐face communication networks. Rather, domestic electrification alleviates operational bottlenecks such as device charging logistics, thereby lowering the friction required to access digital tools. As a result, access to electricity shifts the balance of a farmer′s communication portfolio toward ICT platforms. In nonelectrified households, smallholders are forced to rely almost exclusively on traditional networks out of structural necessity. When electricity becomes available, digital tools assume a more prominent role for time‐sensitive tasks (rapid price tracking), whereas indigenous networks are retained for contextual validation, trust‐building, and peer consultation [83].

In conclusion, age served as a key bifurcating factor in communication channel selection, driving indigenous channel usage while constraining ICT adoption. Beyond age, the simultaneous joint usage of both indigenous and modern ICT channels was strongly driven by credit access, mobile network availability, and extension contact, underscoring the critical role of foundational institutional and social support. In contrast, variables such as education, farm size, farm income, market distance, and electric power supply were primarily decisive in determining modern ICT adoption, demonstrating that socioeconomic status and physical infrastructure remain fundamental prerequisites for technological engagement in agricultural marketing. Other socioeconomic variables specifically gender, nonfarm income, and membership in social or farmers′ organizations did not show statistically significant associations (*p* > 0.10).

### 4.3. Marginal Effects of Factors Influencing Channel Synergy

This section examines the marginal effects (dy/dx) of demographic, socioeconomic, and institutional factors that influence vegetable farmers′ concurrent use of indigenous and ICT communication channels (*P*[*Y*1 = 1, *Y*2 = 1]) for sharing vegetable crop marketing price information. Table 5, presents the marginal effects of these factors on the probability of using combinations of both communication channels. The discussion below focuses on the determinants of joint usage of indigenous and ICT channels for vegetable crop marketing information.

**Table 5:** Marginal effects of determinants on communication channel usage combinations.

Variables	Marginal effect of (both channels)	Marginal effect of (only indigenous channels)	Marginal effect of (only ICT channels)	Marginal effect of (neither channels)
	*P*(*Y*1 = 1, *Y*2 = 1)	*P*(*Y*1 = 1/*Y*2 = 0)	*P*(*Y*1 = 0/*Y*2 = 1)	*P*(*Y*1 = 0/*Y*2 = 0)
	(*p*11)	(*p*10)	(*p*01)	(*p*00)
Age	−0.00540NS	−0.000838	0.00121	0.00502
Gender	0.00108NS	0.0115	−0.0103	−0.00220
Education	0.0129 ^∗∗∗^	−0.0101 ^∗∗∗^	0.0102 ^∗∗∗^	−0.0129 ^∗∗∗^
Farm size	0.0653 ^∗∗∗^	0.0313 ^∗∗∗^	0.0336 ^∗∗∗^	−0.0630 ^∗∗∗^
Family size	0.0136NS	−0.00210	0.000706	−0.0122
Income	0.0139 ^∗∗^	0.121 ^∗∗^	0.120 ^∗∗^	−0.140 ^∗∗^
Nonfarm income	−0.0225NS	−0.0180	0.0181	0.0224
Credit	0.304 ^∗∗∗^	−0.0106 ^∗∗∗^	−0.0167 ^∗∗∗^	−0.277 ^∗∗∗^
Membership	0.0191NS	0.00997	−0.0105	−0.0185
Market distance	−0.00232NS	−0.00372	0.00313	−0.00173
Mobile network	0.262 ^∗∗∗^	−0.0239 ^∗∗∗^	0.00117 ^∗∗∗^	−0.237 ^∗∗∗^
Extension contact	0.0503 ^∗∗∗^	0.00941	0.00410	−0.0449
Electric power	0.133 ^∗∗^	−0.0123 ^∗∗^	0.0224 ^∗∗^	−0.122 ^∗∗^

*Note: Source:* Survey result (2023).  ^∗^, ^∗∗^,  ^∗∗∗^, and NS mean significant at 10%, 5% and 1%, and not significant, respectively.

As presented in Table 5, farmer education level significantly increased the likelihood of jointly utilizing both indigenous and ICT communication channels by (1.29%, *p* < 0.01). Formal education enhances a farmer′s capacity to process complex information, making them more receptive to technological innovations. From a DOI perspective, education lowers the cognitive threshold required for knowledge acquisition. Through an SLT lens, educated smallholders are better positioned to internalize and evaluate technical information presented across diverse media platforms. These findings concur with prior research demonstrating a positive relationship between educational attainment and ICT channel adoption for agricultural market exchanges [84, 85]. Qualitative insights from field interviews strongly support these econometric findings. A 48‐year‐old male extension agent in Damota Kebele, Haramaya Woreda, explained how educational disparities shape channel usage:

Two decades ago, both educated and uneducated farmers relied almost exclusively on indigenous interpersonal networks because ICT access was limited to infrequent, generalized radio broadcasts. Today, while all farmers integrate channels to some extent, educated farmers leverage advanced mobile functions such as mobile banking, SMS, calculators, and direct trader contacts alongside traditional networks. Uneducated farmers primarily use phones for voice calls to peers and rely on family members or educated neighbors for digital functions.

This qualitative evidence highlights how functional literacy acts as a catalyst for digital fluency. Although uneducated farmers utilize basic mobile voice calls to extend their interpersonal networks, formal schooling empowers farmers to utilize advanced digital tools for market transactions, confirming earlier findings by [82].

As presented in Table 5, farm size was a strong positive predictor of communication channel interdependence, increasing the likelihood of jointly utilizing both indigenous and ICT channels by (6.5%, *p* < 0.01). This outcome aligns with the relative advantage construct within the DOI theory. Larger landholdings serve as a proxy for operational scale and financial liquidity, enabling farmers to invest in hardware and experiment with diverse information streams. Furthermore, managing larger commercial volumes requires frequent, multisource market intelligence to mitigate risk and maximize profit margins. This result concurs with prior studies showing that cultivated land area significantly increases the multichannel utilization of mobile phones, radio, and television for agricultural marketing [84]. Qualitative findings from FGDs further illustrate how operational scale drives channel selection. A 42‐year‐old female farmer from Bilisummaa Kebele, Kombolcha Woreda, explained the stark contrast between small‐scale and large‐scale producers:

Smallholders with limited land rely primarily on face‐to‐face exchanges with friends, neighbors, and local traders who come directly to the farm. In contrast, farmers with large landholdings earn higher incomes, allowing them to purchase devices like radios, televisions, and mobile phones to actively track and discuss market prices across multiple channels.

This qualitative narrative underscores how land area and commercial orientation dictate market‐seeking behavior. As noted by [86], larger landholders engage in market transactions more actively and absorb adoption risks more effectively, leading to a broader combination of face‐to‐face interactions, traditional media, and modern ICTs.

As indicated in Table 5, farm income showed a strong positive and statistically significant association with channel interdependence, increasing the likelihood of jointly utilizing both indigenous and ICT communication channels by 1.39 percentage points (*P* = 0.0139, *p* < 0.05). This result directly aligns with the relative advantage construct of the DOI framework, where financial liquidity determines an adopter′s capacity to acquire and maintain innovations. Higher income provides smallholders with the financial means to purchase hardware (e.g., smartphones and radios) and cover recurring service costs (mobile airtime and data packages), enabling them to seamlessly combine digital streams with traditional word‐of‐mouth networks. These findings concur with [86, 87], who demonstrated that higher income smallholders exhibit a significantly greater probability of using mobile phones and radio for agricultural market intelligence.

As shown in Table 5, access to credit services exhibited a strong, highly statistically significant positive association with channel synergy, increasing the likelihood of jointly utilizing both indigenous and ICT communication channels by (30.4%, *p* < 0.001). Access to credit provides essential liquidity, allowing smallholders to overcome capital barriers associated with ICT adoption such as purchasing mobile handsets and paying for airtime or data services while funding participation in local interpersonal networks. This result reinforces the DOI theory regarding financial capital as a prerequisite for technology trialability and adoption, aligning with prior empirical studies [81, 88]. Insights from FGDs further illustrate how credit access alleviates financial constraints for smallholders. A 56‐year‐old male farmer from Tuujii Gabisa Kebele, Haramaya Woreda, described his experience:

In the absence of formal credit institutions in our kebele, I rely on borrowing from family and friends to fund farming inputs. Recently, this informal financial access enabled me to purchase mobile phones for myself and my older son to boost our agricultural productivity before repaying the loan.

This qualitative account demonstrates that credit whether formal or informal alleviates liquidity bottlenecks, empowering smallholders to acquire ICT devices alongside essential agricultural inputs.

Mobile network availability strongly and positively increased the probability of channel complementarity (*P* = 0.262, *p* < 0.001). Farmers residing in networked areas were 26.2% more likely to jointly utilize indigenous and ICT channels for vegetable marketing than those without coverage. Within the DOI framework, cellular infrastructure represents a foundational element of “compatibility,” as network access is a functional prerequisite for digital device viability. Reliable connectivity provides the structural base for farmers to integrate digital tools into traditional information routines, aligning with [63], who emphasized that rural ICT utilization and electronic services depend directly on cellular and grid infrastructure.

Extension contact, contact with agricultural extension agents, had a statistically significant positive effect on channel complementarity, increasing the likelihood of joint channel usage by 5.03%, *p* < 0.01). Extension workers function as classic change agents under DOI theory, providing technical advice and fostering multichannel awareness. This finding agrees with prior research linking extension visits to increased ICT adoption in agriculture [88].

Qualitative insights from FGDs illustrate how extension contact drives multichannel habits. A 30‐year‐old farmer in Kuro Kebele, Haramaya Woreda, explained:

I consult elderly farmers face‐to‐face and phone my peers for local price updates. Crucially, I make frequent visits to the extension office and call agents directly when I need immediate advice. Extension agents serve as key knowledge sources, introducing us to both new production technologies and agricultural ICT tools.

This narrative confirms that extension agents promote technology awareness and multichannel communication, corroborating findings by [89].

For electric power supply, access to a reliable electric power supply significantly increased the probability of joint channel usage by (*P* = 13.3*%*, *p* < 0.05). Electricity constitutes another critical component of DOI compatibility, as device charging and operation are prerequisites for sustained ICT adoption. Lacking electricity creates a severe infrastructure barrier to digital media engagement, whereas electrification promotes device ownership and usage [90]. A 32‐year‐old male farmer in Xiniqee Kebele highlighted this infrastructure linkage:

Having reliable electricity in our kebele allows me to charge my phone, watch TV, and power farm equipment. Electrical access directly enables me to call peers and extension agents to discuss market prices and technology adoption.

As noted by [91], electricity acts as a fundamental driver for rural ICT adoption. Overall, these findings demonstrate that smallholders leverage complementary, multichannel communication strategies to exchange market intelligence; reinforcing research by [92] which showed that exposure to multiple information streams produces more pronounced behavioral changes.

## 5. Conclusion

The findings of this study underscore a fundamental shift in agricultural communication: Smallholder farmers do not operate within an isolated information source but rather navigate a complex, highly synergistic ecosystem of indigenous and ICT channels. Rather than mutually exclusive pathways, indigenous methods and ICT tools function as complementary streams where ICT tools amplify indigenous interpersonal networks rather than replace them. The empirical findings imply that integrated channel usage could potentially enhance price discovery, broaden market reach, and minimize transaction costs for smallholders. Consequently, extension policies that promote combined digital and local information delivery may offer a practical framework for reducing information asymmetry in rural markets.

### 5.1. Summary of Key Drivers of Channel Synergy

Marginal effects analysis from the BVPM demonstrates that the joint usage (*P*(*Y*1 = 1, *Y*2 = 1) of indigenous and ICT channels is governed by foundational institutional, infrastructural, and socioeconomic enablers.

• Institutional and infrastructural catalysts: Access to credit (+30.4%) and mobile network coverage (+26.2%) serve as the strongest determinants of dual‐channel synergy. Credit provides smallholders with the financial liquidity required to acquire mobile handsets and connectivity services, whereas cellular coverage fulfills the basic infrastructural prerequisite for digital functionality. Household electric power supply (+13.3%) further supports device operation, though electrification shifts reliance away from exclusive dependence on nonelectronic indigenous media. Additionally, extension contact (+5.03%) acts as an institutional bridge, facilitating multichannel information seeking.
• Socioeconomic capacities: Larger farm size (+6.53%), higher farm income (+1.39%), and formal education (+1.29%) serve as vital economic and cognitive prerequisites. Landholding size and farm income supply the capital necessary to adopt ICT devices, whereas formal education provides the literacy skills needed to process multichannel market data.
• Generational divergence: Age functions as a bifurcating variable positively predicting indigenous channel usage owing to social trust, community standing, and historical experience but negatively influencing ICT usage because of perceived technological complexity and lower self‐efficacy.

### 5.2. Theoretical Contributions

This study contributes to agricultural communication literature by conceptualizing ICT tools as conceptual *amplifiers* rather than *replacements* of indigenous systems:

• DOI perspective: Cellular coverage and domestic electrification provide the necessary *compatibility* for technology adoption, whereas farm size and income establish the *relative advantage* that enables smallholders to invest in ICT assets.
• SLT perspective: Educational attainment builds cognitive self‐efficacy for processing complex market inputs across multiple media, whereas younger farmers demonstrate higher self‐efficacy in adopting digital tools compared with older cohorts.

### 5.3. Policy and Practical Implications

These empirical insights offer actionable guidance for agricultural extension systems and policy design:

• For extension officers: Agricultural extension must transition away from single‐medium communication strategies. Extension agents should leverage ICT platforms (e.g., SMS alerts and market price applications) to complement face‐to‐face consultations and group gatherings, using mobile tools to amplify traditional word‐of‐mouth networks over wider geographical distances.
• For policymakers: Public infrastructure investments must prioritize expanding foundational rural connectivity, rural grid electrification, and accessible microcredit schemes. Without these primary enablers, digital agricultural initiatives will fail to achieve widespread adoption.

### 5.4. Addressing Equity and ICT Inclusion

The strong influence of wealth, formal education, and age reveals a critical equity gap: Resource‐constrained, illiterate, and older smallholders face heightened risks of ICT exclusion. To prevent widening socioeconomic divides, policymakers and development partners should implement targeted inclusive strategies, including the following:

• Subsidized basic mobile handsets for low‐income farming households.
• Tailored digital literacy and skills training for older rural cohorts.
• Community‐based media hubs and voice‐based/local‐language ICT services designed for low‐literacy users.

## 6. Recommendations

The significant positive correlation (rho = 0.498) and substantial marginal effects of institutional enablers demonstrate that single‐medium ICT strategies are insufficient for smallholder systems. To optimize vegetable marketing information systems and maximize channel synergy, the following targeted, multistakeholder interventions are recommended.

Financial institutions and microfinance agencies should transition toward bundled credit products that combine traditional input loans with ICT financing (smartphone lease‐to‐own schemes, device insurance, and subsidized data bundles).

• Key implementation actors: microfinance institutions (MFIs), commercial banks in Ethiopia, and agricultural cooperatives.
• Impact and feasibility: Given that credit access yields the highest marginal effect (30.4%) on channel synergy, removing upfront hardware costs enables resource‐poor farmers to access real‐time market data alongside local networks.

The government must prioritize expanding cellular coverage (+26.2% marginal effect) and grid electrification (+13.3%) in major vegetable‐producing corridors. In off‐grid or power‐constrained rural kebeles, local authorities and development partners should establish solar‐powered community ICT hubs equipped with multidevice charging kiosks and public market information displays.

• Key implementation actors: Ministry of Innovation and Technology (MInT), Ministry of Water and Energy (MoWE), Ethio‐Telecom, Regional Energy Bureaus, and local youth microenterprises.
• Cost‐feasibility and implementation blueprint: Rather than requiring heavy capital expenditures, small‐scale solar charging kiosks and digital display hubs (equipped with 300–500 W solar panel arrays, lithium battery storage, multiport charging stations, and a digital price board) require an estimated initial investment of 1200 to 1800 USD per kebele hub. Existing operational models such as the solar‐powered youth enterprise kiosks piloted by the Solar Energy Development Association (SEDA) and the UNICEF‐supported rural digital kiosks in Ethiopia demonstrate that operational costs are fully covered through microfees charged for mobile phone powering (e.g., 5–10 ETB per charge). Managed by local youth cooperatives, these hubs achieve self‐sustainability within 6–8 months while eliminating battery charging friction for off‐grid farmers.

Agricultural extension services (+5.03% marginal effect) should transition from top‐down technology promotion toward a formally institutionalized hybrid extension model. The hybrid extension model is a dual‐channel advisory framework that systematically integrates ICT sourced, real‐time market intelligence with indigenous, trusted interpersonal extension delivery mechanisms (development agents, model farmers, and village group gatherings).

• Key implementation actors: Ministry of Agriculture (MoA), Oromia Regional Bureau of Agriculture, Haramaya University Extension Outreach Directorate, and Agricultural TVET Colleges.

Proposed pilot program design (3‐phase implementation):

▪ Phase 1: Agent digital capability and group creation (Months 1–3): Equip local development agents (DAs) with basic smart devices and train them to curate daily price trends via digital platforms (e.g., Telegram channels or national market databases). DAs establish localized voice/messaging groups aligned with existing Kebele Development Committees.
▪ Phase 2: Dual‐channel delivery integration (Months 4–9): During weekly face‐to‐face farmer training sessions and field days, DAs crossvalidate digital market data with local indigenous insights gathered from community elders and lead traders, broadcasting weekly validated summaries via local radio and group messaging.
▪ Phase 3: Feedback and peer mentorship evaluation (Months 10–12): Evaluate adoption metrics, price realization, and user satisfaction among older versus younger farmers to refine advisory content.

Software developers and AgTech startups should design “socially validated” ICT platforms tailored to older and low‐literacy users. Applications should incorporate local‐language voice interfaces, audio/video messaging, interactive voice response (IVR) options, and peer‐to‐peer price verification features.

• Key implementation actors: AgTech developers, telecommunication companies, and agricultural research institutes.
• Impact and feasibility: By mirroring oral and communal communication habits within digital interfaces, developers reduce perceived technology complexity (DOI) and boost user self‐efficacy SLT, bridging the generational digital divide for older smallholders.

Policymakers and local agricultural offices should actively promote and strengthen vegetable marketing cooperatives. By pooling smallholder output and capital, cooperatives can achieve the relative advantage enjoyed by larger farms (6.53% marginal effect), enabling collective investment in shared market tracking tools, bulk mobile data, and organized trade networks.

• Key implementation actors: cooperative promotion agencies, local agricultural offices, and farmer cooperatives.
• Impact and feasibility: Resource pooling ensures that smallholders with smaller landholdings and lower farm incomes (1.39% marginal effect) can access digital market intelligence without incurring prohibitive individual expenses.

To ensure equity and combat digital exclusion among low‐income, female, and small‐scale vegetable producers, nongovernmental organizations and community structures must deploy targeted support programs.

• Key implementation actors (specific to East Hararghe Zone): International and regional NGOs operating directly within the East Hararghe Zone such as CARE Ethiopia (via its Livelihoods and Microfinance Interventions in Eastern Oromia), Catholic Relief Services (CRS Ethiopia), Self Help Africa, and the Hararghe Catholic Secretariat (HCS), alongside local community‐based organizations (Iddirs and primary cooperatives).
• Actionable zonal strategy: These active organizations should integrate hardware matching‐grants (50% subsidized basic phones for female‐headed and resource‐poor households) directly into their ongoing food security and rural livelihood projects in East Hararghe. Concurrently, cooperatives and Iddirs should formalize “digital information circles,” wherein tech‐literate younger farmers act as designated peer mentors, assisting older or illiterate group members in interpreting SMS price alerts and conducting mobile financial transactions.

## 7. Limitations of the Study

The empirical findings of this research should be interpreted within the context of several inherent methodological, geographic, and contextual limitations:

• Geographic scope and regional generalizability: The study was geographically restricted to the Haramaya and Kombolcha woredas within the East Hararghe Zone of Oromia Region. Because these woredas were specifically selected because of their high vegetable production potential, established irrigation access, and relative proximity to major urban market centers (Harar and Dire Dawa), the results may not fully generalize to remote, arid, subsistence‐oriented, or less commercially active agrarian regions of Ethiopia.
• Methodological and cross‐sectional design: The reliance on a cross‐sectional survey design captures farmer communication decisions and information‐seeking behavior at a single point in time (June–September 2023). Consequently, the static nature of the data cannot capture longitudinal changes over time, long‐term technological adoption trajectories, or seasonal fluctuations in communication dynamics across different cropping and marketing cycles.
• Methodological exclusion of correlated variables: Because of diagnostic indications of moderate‐to‐high multicollinearity (VIF = 5.03), the variable access to market information was removed prior to running the final BVPM. Although sensitivity tests confirmed that its structural effects were adequately captured by retained institutional indicators (such as extension contact, organizational membership, and mobile network coverage), its formal removal means that direct marginal effects for market information access per se could not be isolated independently within the econometric model.
• Deliberate tool scope and digital platform boundaries: The analytical scope of ICT tools was intentionally bounded to basic, widely accessible hardware and media—specifically radio, television, and mobile phones (voice/SMS)—within the vegetable value chain. This focus represents a deliberate scope choice driven by the baseline technology adoption levels in rural East Hararghe rather than an empirical oversight. Consequently, the study does not evaluate advanced or emerging digital platforms, such as specialized smartphone applications, web‐based agtech portals, or social media trade channels (e.g., Telegram groups or WhatsApp market networks), which may exhibit distinct adoption mechanics among more commercialized producers.
• Gender imbalance and sample generalizability: The final survey sample was predominantly male‐headed (85.5% male vs. 14.5% female). This distribution was an incidental outcome of sampling head‐of‐household registries maintained at local kebele administrative offices, where land ownership and primary farming decisions are culturally and legally registered to male household heads. Although this reflects the prevailing demographic structure of landholding decision‐makers in the target kebeles, it presents a limitation regarding the generalizability of gender‐specific findings. Institutional and social barriers such as unequal resource access, restricted extension outreach, and lower ICT ownership affect female smallholders uniquely, requiring caution when extending these econometric conclusions to female‐headed households or joint gender decision‐making dynamics across the agricultural sector.
• Data accuracy and recall bias: Data collection was constrained by smallholders′ lack of formal record‐keeping and reluctance to disclose exact financial earnings. To address this, the study relied on self‐reported harvest volumes, expenditure recall, and financial figures. This approach introduces potential recall bias, as farmers may not have perfectly remembered precise seasonal yields, input costs, or fluctuating price realizations from prior marketing periods.

## 8. Suggestions for Future Research

To build upon the empirical and theoretical insights gained from this study, future research should expand the geographical, technological, and demographic scope of agricultural communication inquiry:

• Longitudinal panel designs: Researchers are encouraged to transition from static cross‐sectional snapshots toward longitudinal panel designs that track communication patterns over multiple cropping seasons. Multiyear tracking is essential for capturing seasonal fluctuations in market information demand, distinguishing temporary tool trialability from sustained ICT adoption, and establishing clear causal pathways over time.
• Agroecological and regional comparative studies: Given this study′s focus on the commercial vegetable corridors of the East Hararghe Zone, future research should conduct comparative studies across diverse Ethiopian agroecological zones (e.g., pastoralist lowlands, highland cereal‐based systems, and remote subsistence districts) and alternative crop types (such as staple grains or coffee). Broadening spatial, cultural, and commodity coverage will test the generalizability of the channel synergy concept under varying market structures, infrastructure baselines, and regional social dynamics.
• Investigating emerging “hybrid” digital tools: Future research should examine the rapid emergence of social media platforms (such as Telegram trade groups and Facebook agricultural communities) and voice‐based ICTs ([IVR] systems and radio call‐in programs). These platforms act as “hybrid tools” that blend digital reach with oral, conversational, and communal traditions effectively blurring the boundary between traditional indigenous communication and modern ICT tools. Evaluating how smallholders navigate these hybrid spaces will provide a forward‐looking perspective on the modernizing rural information ecosystem.
• Enhanced financial data verification: To address recall bias, subsequent inquiries should employ more rigorous financial data verification methods such as real‐time digital sales logging, mobile money transaction logs (e.g., Telebirr), or direct triangulation with market broker ledgers. Improving income and yield data precision will refine wealth‐based econometric adoption models.
• Digital equity and inclusivity frameworks: Future research must explicitly incorporate digital equity and gender frameworks to analyze the structural barriers facing marginalized farming cohorts, particularly resource‐poor, low‐literacy, female, and older producers. Prioritizing digital equity ensures that future AgTech innovations and national extension policies cater to the specific needs of vulnerable smallholders, preventing technological transformation from exacerbating rural socioeconomic divides.

## Author Contributions

Getahun Milion Agago: Conceptualization, data curation, formal analysis, investigation, methodology, validation, writing—original draft, and writing—review and editing. Getachew Shambel Endris: Conceptualization, project administration, supervision, validation, and writing—review and editing. Chanyalew Seyoum Aweke: Conceptualization, project administration, supervision, validation, and writing—review and editing.

## Funding

No funding was received for this manuscript.

## Conflicts of Interest

The authors declare no conflicts of interest.

## Data Availability

The data that support the findings of this study are available from the corresponding author upon reasonable request.
